# PGC-1α activator ZLN005 promotes maturation of cardiomyocytes derived from human embryonic stem cells

**DOI:** 10.18632/aging.103088

**Published:** 2020-04-28

**Authors:** Yanping Liu, Huajun Bai, Fengfeng Guo, Phung N. Thai, Xiaoling Luo, Peng Zhang, Chunli Yang, Xueqin Feng, Dan Zhu, Jun Guo, Ping Liang, Zhice Xu, Huangtian Yang, Xiyuan Lu

**Affiliations:** 1 Institute for Fetology, First Hospital of Soochow University, Suzhou, China; 2 CAS Key Laboratory of Tissue Microenvironment and Tumor, Laboratory of Molecular Cardiology, Shanghai Institute of Nutrition and Health, University of Chinese Academy of Sciences (CAS), CAS, Shanghai, China; 3 Key Laboratory of Combined Multi-organ Transplantation, Ministry of Public Health, The First Affiliated Hospital, Zhejiang University, Hangzhou, China; 4 Institute of Translational Medicine, Zhejiang University, Hangzhou, China; 5 Department of Internal Medicine, University of California Davis, Davis, CA 95616, USA; 6 Institute for Stem Cell and Regeneration, CAS, Beijing, China

**Keywords:** embryonic stem cells, cardiomyocyte maturation, peroxisome proliferator-activated receptor gamma coactivator 1α, ZLN005, metabolism

## Abstract

Human pluripotent stem cell-derived cardiomyocytes (hPSC-CMs) have great potential in biomedical applications. However, the immature state of cardiomyocytes obtained using existing protocols limits the application of hPSC-CMs. Unlike adult cardiac myocytes, hPSC-CMs generate ATP through an immature metabolic pathway—aerobic glycolysis, instead of mitochondrial oxidative phosphorylation (OXPHOS). Hence, metabolic switching is critical for functional maturation in hPSC-CMs. Peroxisome proliferator-activated receptor gamma coactivator 1α (PGC-1α) is a key regulator of mitochondrial biogenesis and metabolism, which may help promote cardiac maturation during development. In this study, we investigated the effects of PGC-1α and its activator ZLN005 on the maturation of human embryonic stem cell-derived cardiomyocyte (hESC-CM). hESC-CMs were generated using a chemically defined differentiation protocol and supplemented with either ZLN005 or DMSO (control) on differentiating days 10 to 12. Biological assays were then performed around day 30. ZLN005 treatment upregulated the expressions of PGC-1α and mitochondrial function-related genes in hESC-CMs and induced more mature energy metabolism compared with the control group. In addition, ZLN005 treatment increased cell sarcomere length, improved cell calcium handling, and enhanced intercellular connectivity. These findings support an effective approach to promote hESC-CM maturation, which is critical for the application of hESC-CM in disease modeling, drug screening, and engineering cardiac tissue.

## INTRODUCTION

Human cardiovascular diseases continue to cause major health and economic burden worldwide [1, 2]. Cardiomyocytes differentiated from human pluripotent stem cells (hPSC-CMs), including both embryonic stem cell-derived cardiomyocytes (hESC-CMs) and induced pluripotent stem cell-derived cardiomyocytes (hiPSC-CMs), provide an enormous potential for the development of tissue engineering, drug screening, and cardiac disease modeling platforms [3–5]. Acquiring mature hiPSC-CMs, however, remains a significant and challenging problem. The immaturity of hiPSC-CMs—manifested as disorganized sarcomere structures, underdeveloped calcium handling, weak excitability and contractility, and slow action potential conduction—limits their potential impact [6–8]. Therefore, strategies that help promote the maturation of hPSC-CMs are indispensable for the application of hPSC-CMs. Several approaches have been reported to enhance hPSC-CMs’ maturation, such as long-term culture, bioelectrical stimulation, mechanical stretch, biochemical stimulation, and the incorporation of CMs into 3D tissue constructs [9–13]; however, since these methods are time consuming, expensive, and technically challenging, they are not suitable for widespread adoption. Here, we report a rapid, simple, and affordable method to promote the maturation of hPSC-CMs.

*In vivo*, cardiomyocytes undergo multiple adaptive, structural, functional, and metabolic alterations during maturation, while hPSC-CMs fail to fully experience these developmental processes [14]. The mitochondrial structure and function change drastically during cardiac development [15, 16]. A transition in energy metabolism is implicated in cardiogenesis and maturation, including mitochondrial biogenesis, fuel shift, mitochondrial quantity, and morphology [17, 18]. Mitochondria in immature cardiomyocytes occupy a small fraction of cell volume, and distribute irregularly in the cytoplasm [19, 20], whereas mitochondria in mature cardiomyocytes occupy ~20–40% of the myocyte volume, and have extensive surface area provided by densely packed cristae [19, 20]. During development, fetal cardiomyocytes reside in a hypoxic environment and mainly rely on glycolysis to generate ATP. On the contrary, adult cardiomyocytes mostly depend on mitochondrial oxidative phosphorylation for ATP generation, especially through fatty acid oxidation (FAO) [8, 21, 22]. The adult heart is a high energy consumption organ and always changes its workload, so it is critical for the heart to adjust its energy output to properly support contraction. Hence, strategies promoting metabolic maturation and metabolic-contraction coupling are promising to promote cardiomyocytes’ maturation [6, 18, 23].

Cardiac energy metabolism is regulated by several molecular pathways during cardiac development. The peroxisome proliferator-activated receptor gamma coactivator 1 (PGC-1) family consists of three isotypes—PGC-1α, PGC-1β, and PGC-1-related coactivator (PRC)—that function as transcriptional co-activators [24]. PGC-1s activate a variety of coactivated genes, including estrogen-related receptors (ERRs), nuclear respiratory factors (NRFs), and peroxisome-proliferating receptors (PPARs), and they can be regulated at both the transcriptional and post-translational levels [25]. Of all the isoforms, PGC-1α is a key regulator of mitochondrial biogenesis and metabolism, and it is highly expressed in organs with high energy oxidative capacity, such as the heart [26]. By modulating the binding activity of different transcription factors, PGC-1α upregulates the expression of various genes involved in diverse metabolic pathways, including fatty acid oxidation, glycolysis, fatty acid synthesis, and mitochondrial biogenesis [27–29]. In addition, it has been reported that PGC-1α is repressed in cardiac hypertrophy, which accompanied a decrease in fatty acid β-oxidation and mitochondrial oxidative capacity [27, 30, 31]. Conversely, PGC-1α overexpression induced the expression of a number of coactivated genes (ERRs, PPARs, and NRFs), which virtually enhanced all aspects of mitochondrial biogenesis and energy metabolism [24, 32, 33]. Collectively, PGC-1α can function as a metabolic regulator, which makes it an attractive target to promote the maturation of cardiomyocytes.

Although activation of PGC-1α can occur through the β-adrenergic receptor (β-AR)/3'-5'-cyclic adenosine monophosphate (cAMP) and AMP-activated protein kinase (AMPK) pathways [33, 34], more recent studies have utilized a small, benzimidazole compound ZLN005 to induce PGC-1α expression [35–41]. Hence, the application of ZLN005 to activate PGC-1α might be an effective approach to promote cardiomyocyte maturation.

In this study, we tested the hypothesis that increasing PGC-1α pathway with the small compound ZLN005 would enhance mitochondrial metabolism, ensure proper coupling of energy production and force generation, and improve the maturation of hESC-CMs. To this end, the early stage hESC-CMs (day 10) were treated with ZLN005 for 48h and then subjected to molecular and functional analysis at day 30. Our results show the underlying mechanism controlling the metabolic maturation of stem cell–derived cardiomyocytes and demonstrate the application of ZLN005 as an effective and convenient approach in enhancing various aspects hPSC-CMs maturation.

## RESULTS

### Expression profile of PGC-1α during cardiomyocyte differentiation of hESCs

**Figure 1:**
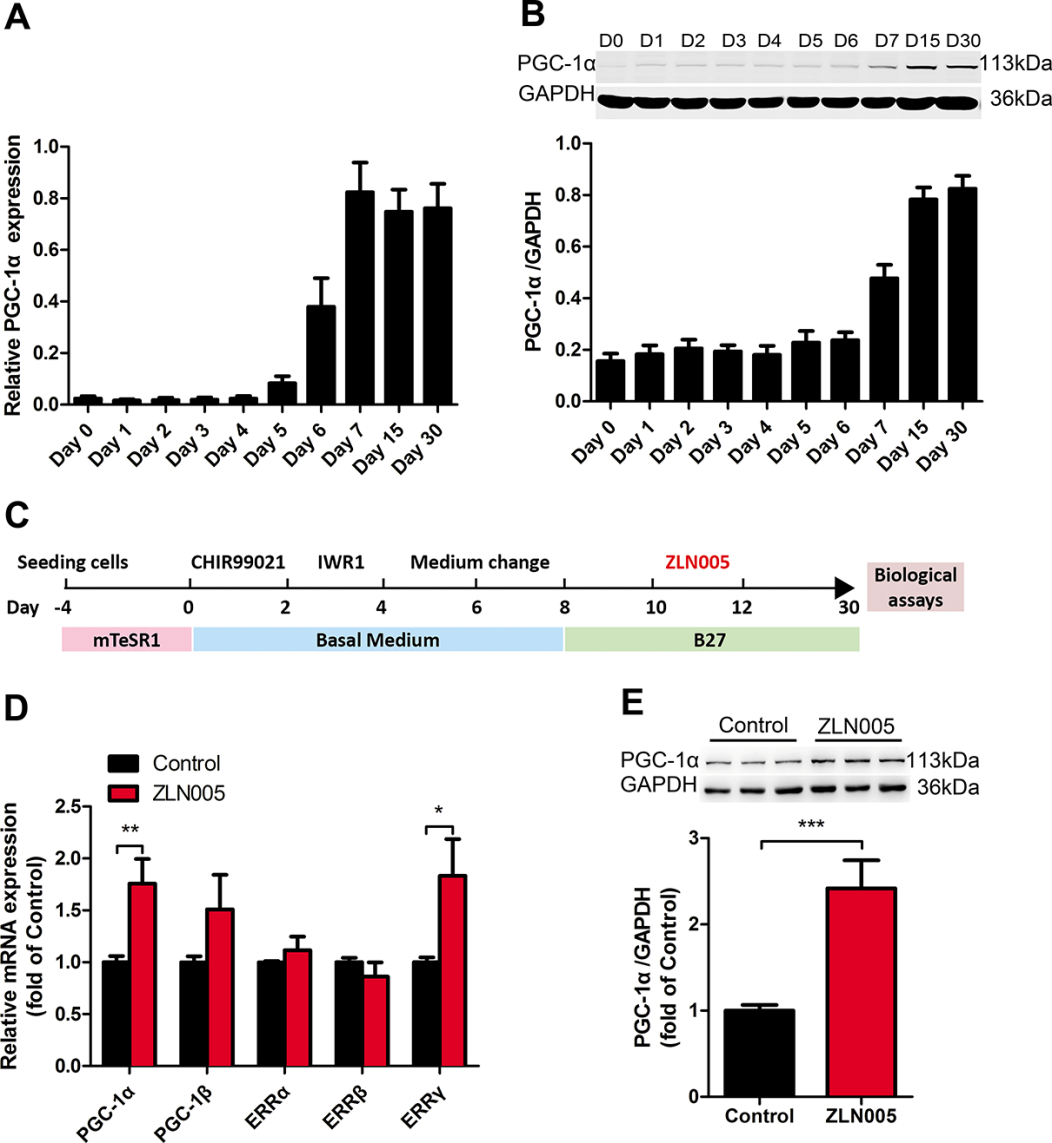
**The expression of PGC-1α was upregulated during cardiomyocyte differentiation; ZLN005 increased PGC-1α mRNA and protein level in hESC-CMs.** (**A**) The relative mRNA and (**B**) protein expression of PGC-α during cardiomyocyte differentiation (mRNA, n=7; protein, n=5). (**C**) Schematic representation of the experimental schedule including hESC culture, cardiomyocyte differentiation, culture and treatment. (**D**) Effect of ZLN005 on mRNA levels (n=6). (**E**) Effect of ZLN005 on PGC-α protein expression (n=12).

The PGC-1 family consists of a group of transcriptional coregulators that coordinates mitochondrial biogenesis and is highly expressed in cardiac tissue [26]. To investigate which PGC-1 family member may facilitate hPSC-CMs maturation, we examined the expression profile of PGC-1 family members during cardiac differentiation of hESCs. As shown in Figure 1A, the expression of PGC-1α mRNA was significantly upregulated from cardiac differentiation of hPSCs at day 5 and day 6, reached the peak at differentiation day 7, and then sustained high expression during days 15-30. In addition, the protein level of PGC-1α also dramatically increased from differentiation day 7 (Figure 1B). However, there was no significant change in the expression of PGC-1β mRNA during CM differentiation and maturation (Supplementary Figure 1B). Hence, manipulating PGC-1α might be an effective approach to promote hPSC-CM maturation.

### ZLN005 treatment upregulated the levels of PGC-1α mRNA and protein in hESC-CMs

It has been shown that treatment with 10 μM ZLN005 for 48h can effectively increase the expression of PGC-1α in skeletal and cardiac myocytes [35, 37, 38, 41]. In this study, to test whether the biological effects of ZLN005 were relevant with PGC-1α expression in hESC-CMs, we generated hPSC-CMs and treated hESC-CMs with ZLN005 (10 μM) from day 10 to day 12, as depicted in Figure 1C. In brief, early hESC-CMs (day 10) were treated with DMSO (control) or ZLN005 at a final concentration of 10 μM for 48h, and then subjected to biological and functional analyses at day 30. As shown in Figure 1D, 10 μM ZLN005 caused a 1.7 fold increase of PGC-1α mRNA levels over the control after 48h of treatment. PGC-1α protein expression was significantly elevated in the ZLN005-treated groups compared with control groups, as evident in Figure 1E. These results demonstrate that ZLN005 increases the expression of PGC-1α in hESC-CMs at both the transcript and protein levels.

### ZLN005 improved mitochondrial maturation of hESC-CMs

**Figure 2:**
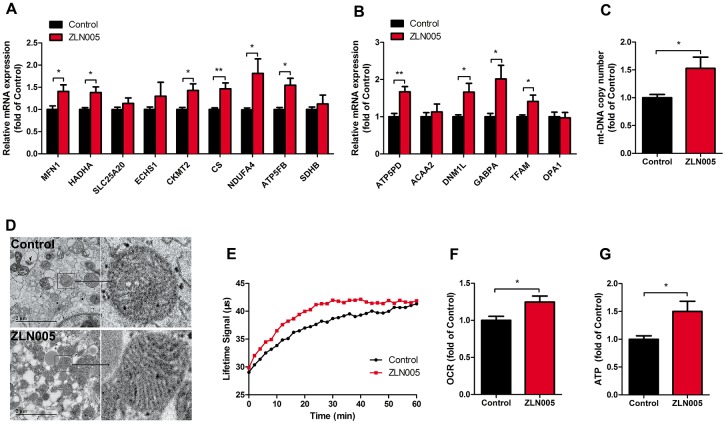
**ZLN005 improved mitochondrial maturation of hESC-CMs.** (**A**) qRT-PCR analysis of mitochondrial oxidative phosphorylation markers in hESC-CMs (n=7). (**B**) Representative lifetime profiles from control (black trace) and ZLN005-treated (red trace) hESC-CMs. (**C**) Basal oxygen consumption rates (lifetime slope) in control and ZLN005-treated hESC-CMs (n=8). (**D**) qRT-PCR analysis of mitochondrial biogenesis markers in hESC-CMs (n=7). (**E**) Mitochondrial DNA content, as determined by qRT-PCR using primers for mt-ND1 normalized to housekeeping gene β-actin (n=8). (**F**) Transmission electron microscopy (TEM) pictures in control and ZLN005-treated hESC-CMs. Scale bar, 2μm. (**G**) Basal ATP levels in hESC-CMs (n=6). A ratiometric analysis was performed to determine changes in the Lifetime fluorescence signal: Lifetime (μs) [T] = (D2-D1)/ln(W1/W2), where D is delay; W is fluorescence window value at each time point.

As a coactivator of the PGC-1 family, the estrogen related receptors (ERRs) play a key role in regulating genes involved in biogenesis, respiratory function, and mitochondrial fatty acid oxidation [42]. We therefore examined the expression levels of PGC1 family and ER family. After treatment with ZLN005, the expression of PGC-1α and ERRγ mRNA was significantly upregulated (Figure 1D). In addition, qRT-PCR analysis revealed that gene expression for mitochondrial biogenesis (GABPA, TFAM), mitochondrial OXPHOS (NRF1, CS (TCA cycle), NDUFA4 (i), SDHB (II), COX5a (IV), ATP5b (V)), and fatty acid β-oxidation (CPT1b, CPT2, SLC25a20, ACADM, ESCHS1, HADHA) markers were significantly upregulated in the hESC-CMs following ZLN0025 treatment relative to the control (Figure 2A, 2B). To determine whether the ZLN005 treatment had effects on the mitochondrial DNA content, we examined the mtDNA copy number. qPCR analysis revealed the mtDNA copy number was 1.5-fold higher in the ZLN005-treated hESC-CMs than in the control (Figure 2C). Transmission electron microscopy analysis showed that the mitochondrial number in the ZLN005-treated hESC-CMs was significantly increased (Figure 2D) and ZLN005-treated hESC-CMs contained more mitochondrial cristae formation than those in the controls (Figure 2D). To characterize mitochondrial energy metabolism, we further analyzed the oxygen consumption rate (OCR) values and ATP production between ZLN0025 treatment and control hESC-CMs. As shown in Figure 2E–2G, the cardiomyocytes treated with ZLN005 had significantly higher basal OCR and levels of ATP per cell compared with the controls.

### ZLN005 improved structural maturation of hESC-CMs

**Figure 3:**
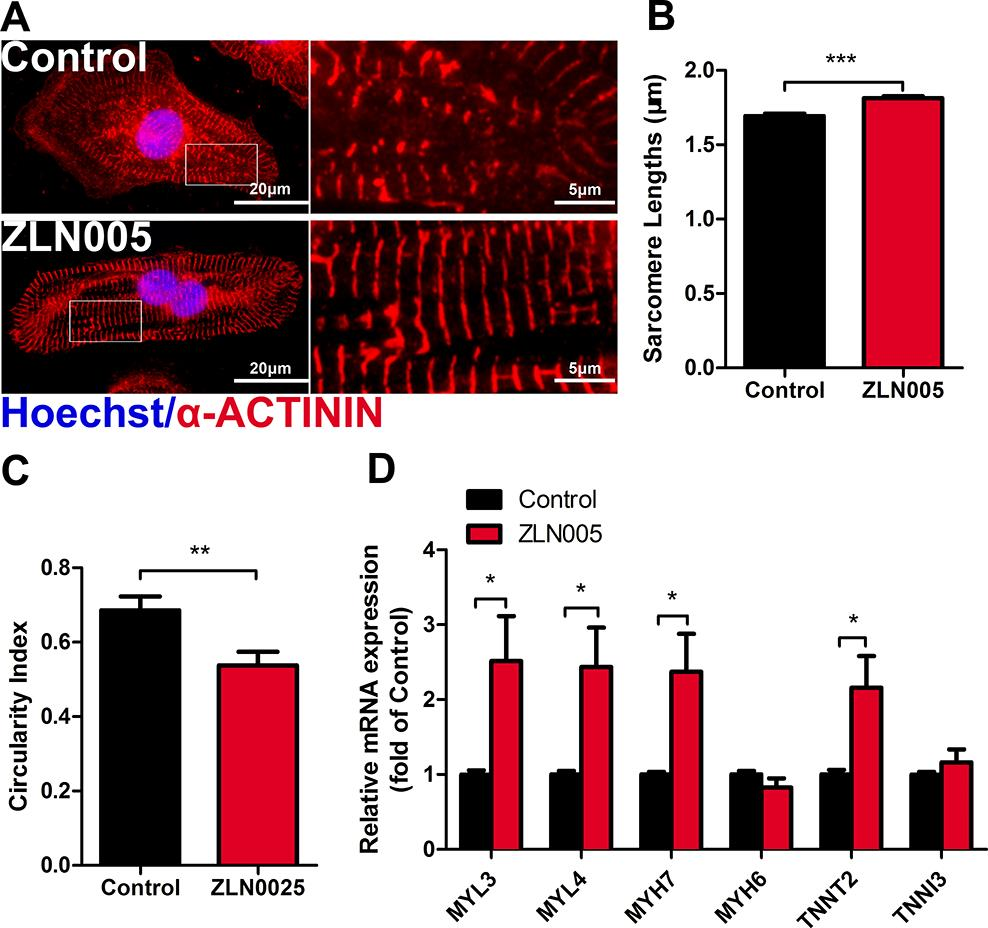
**ZLN005 improved cardiac structural maturation.** (**A**) Representative immunostaining of α-ACTININ (red) and Hoechst 33342 (blue) in control- or ZLN005-treated hESC-CMs. Scale bar, 20μm and 5μm. (**B** and **C**) ZLN005-treated hESC-CMs showed significant increase in sarcomere length and a decrease in circularity index compared to control. n=20-60 cells per condition. (**D**) qRT-PCR analysis of cardiac structural maturation markers in control and ZLN005-treated hESC-CMs (n=6).

Immunostaining results of α-actinin showed clear sarcomere structures in both ZLN005-treated and the control groups (Figure 3A); however, the hESC-CMs treated with ZLN005 had longer sarcomeres (1.69 ± 0.02 vs 1.81 ± 0.01 μm, *P*<0.001) (Figure 3B) and a lower circularity index (0.69 ± 0.04 vs 0.54 ± 0.04, *P*=0.007) (Figure 3C) than those of control ones. Additionally, qRT-PCR results showed that the structural genes (MYL3, MYL4, MYH7, TNNT2) were upregulated in the ZLN005-treated cardiomyocytes (Figure 3D). Taken together, these results demonstrate that ZLN005-treated hESC-CMs exhibit a more mature morphology.

### ZLN005-treated hESC-CMs show improved electrical activity and synchronicity

**Figure 4:**
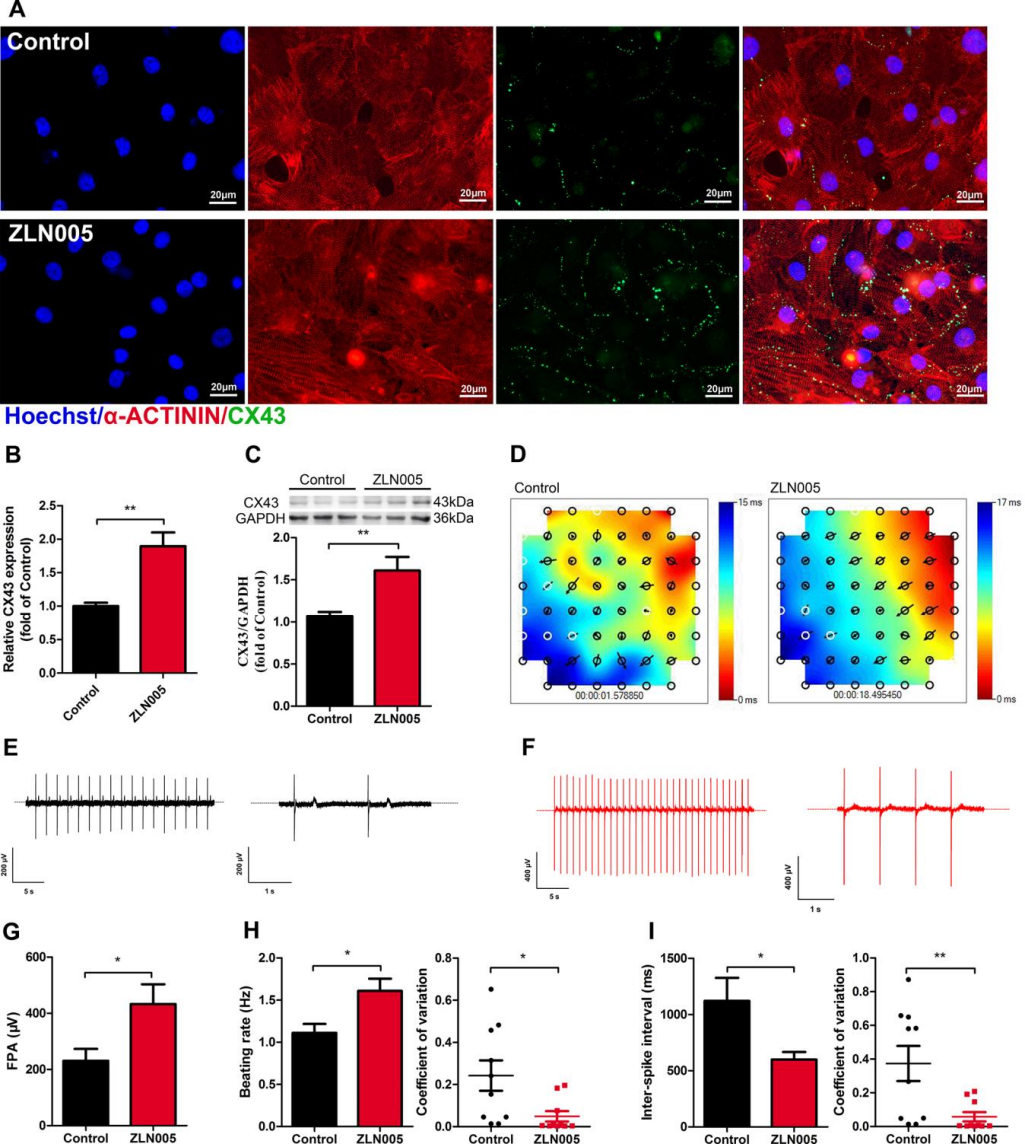
**ZLN005 treatment increased expression of Connexin 43 (CX43) and improved electrical activity in hESC-CMs.** (**A**) Representative immunostaining images of CX43 (green) in control and ZLN005-treated hESC-CMs. Hoechst 33342 (blue) and α-ACTININ (red) were also co-stained in the same cells. Scale bar, 20 μm. (**B**) qRT-PCR analysis of CX43 expression (n=6). (**C**) Representative Western blot and quantification showed up-regulation of CX43 protein expression with ZLN005 treatment (n=6-9). (**D**) Representative color map of electrical signal propagation from control and ZLN005-treated hESC-CMs. The color map shows that the electrical signal is initiated at the upper right corner (red) and is propagated to the bottom left corner (blue). The black arrows indicate the direction of the instantaneously local electrical propagation. (**E**, **F**) Representative field potential tracings recorded from control (black) and ZLN005-treated (red) hESC-CMs, respectively. (**G**) Bar graph to compare field potential amplitude (FPA) between control and ZLN005-treated hESC-CMs (n=10). (**H**) Bar graphs to compare beating rate (left) and coefficient of variation (right) between control and ZLN005-treated hESC-CMs (n=10). (**I**) Bar graphs to compare inter-spike interval (left) and coefficient of variation (right) between control and ZLN005-treated hESC-CMs (n=10).

Gap junction morphology is an important parameter for evaluating the maturation state of hPSC-CMs. Connexin 43 (CX43), a major component of gap junctions, has fundamental roles in excitable tissues by facilitating rapid transmission of action potentials between adjacent cells. We therefore tested whether ZLN005 treatment affected CX43 expression. As shown in the supplementary movies (Supplementary Movie 1–4), ZLN005-treated hESC-CMs exhibited more homogeneous, spontaneous beating rhythm and electrical propagation. Immunostaining results showed that ZLN005 treatment upregulated the density of CX43 (Figure 4A). The enhanced levels of CX43 in the ZLN005-treated hESC-CMs were confirmed by qRT-PCR (Figure 4B) and Western blot (Figure 4C). Furthermore, electrical propagation and activity of CMs were evaluated by measuring surface potentials on Microelectrode arrays (MEAs). The color map represents a snapshot of the electrical propagation in the monolayer where the direction of the arrow indicates the direction of local electrical propagation, suggesting that hESC-CMs developed a more homogeneity electrical propagation due to ZLN005 treatment (Figure 4D). Representative sample traces of spontaneous beating in control (black) and ZLN005 treated (red) CMs are shown in Figure 4D, 4E, respectively. Detailed analyses revealed that the ZLN005-treated CMs exhibited greater field potential amplitude (FPA) (Figure 4G) than those in the control cells. Further analyses showed that ZLN005 treatment significantly increased the frequency of spontaneous beatings (1.11 ± 0.21 Hz vs 1.61 ± 0.12 Hz) (Figure 4H) and decreased the inter-spike interval time (1122.1 ± 205.1 ms vs 598.8 ± 68.42 ms). In addition, the electrical activity exhibited higher regularity and reduced variability in both parameters as evident by the reduced coefficient of variation, indicating a high degree of temporally synchronized depolarization and strong electrical coupling across the cell monolayer. These data demonstrate that ZLN005 treatment significantly improves electrical activity and synchronicity of hESC-CMs.

### ZLN005-treated hESC-CMs exhibited more negative resting membrane potential

**Figure 5:**
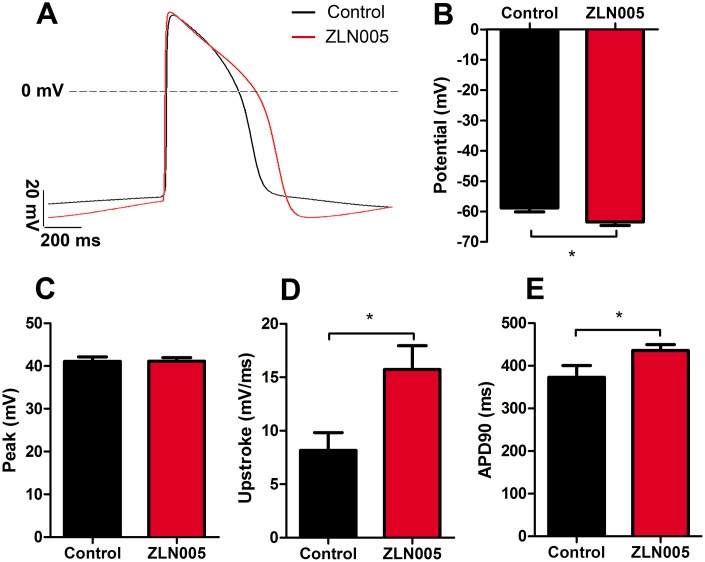
**ZLN005-treated hESC-CMs exhibited more negative resting membrane potential compared with control.** (**A**) Representative spontaneous action potential traces from control (red trace) and ZLN005-treated (black trace) hESC-CMs. (**B**–**E**) Action potential properties of control and ZLN005-treated hESC-CMs: the resting membrane potentials (**B**), peak amplitude (**C**), velocity of upstroke (**D**), action potential durations at 90% repolarization (APD90) (**E**) n=24-54 cells for each group.

To further determine the maturity of electrical properties of the hESC-CMs, we performed electrophysiological assessments (representative spontaneous action potential traces, Figure 5A) and found that ZLN005-treated hESC-CMs had a more negative resting membrane potential compared with cardiomyocytes from the control group (−58.83 ± 1.28 vs. −63.47 ± 1.14 mV, *P*=0.01, Figure 5B) and no significant differences in peak amplitude (Figure 5C). The action potential duration at 90% repolarization (APD90) and the maximum upstroke velocity were significantly higher in the ZLN005 treated CMs (Figure 5D, 5E).

### ZLN005-treated hESC-CMs exhibited more mature calcium transient kinetics

**Figure 6:**
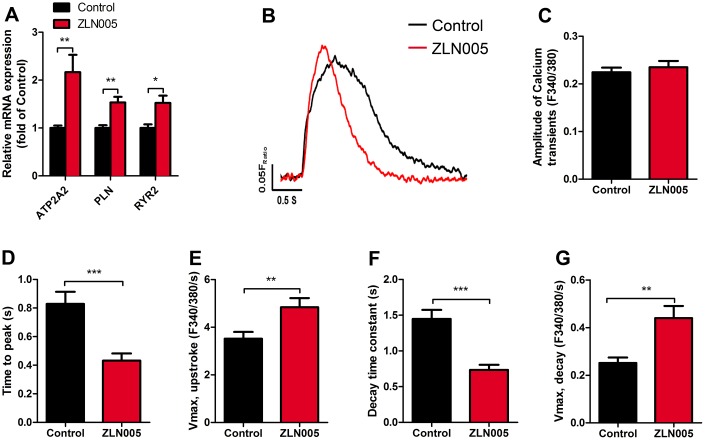
**ZLN005-treated hESC-CMs displayed an increase in calcium signaling and kinetics compared with control.** (**A**) qRT-PCR analysis of cardiac calcium handling markers in control and ZLN005-treated hESC-CMs (n=6). (**B**) Representative intracellular Ca^2+^ transients from control (red trace) and ZLN005-treated (black trace) hESC-CMs. Calcium transients were evaluated by loading the hESC-CMs with fura-2 AM. (**C**–**G**) Ca^2+^ transients properties of control and ZLN005-treated ESC-CMs: The amplitude of Ca^2+^ transient (**C**) time to peak (**D**) maximal velocity of upstroke (**E**) decay time (**F**) maximal velocity of decay (**G**). n=15-20 cells for each group.

Since Ca^2+^ transients are essential for the regulation of excitation-contraction coupling in CMs, we next determined the effects of ZLN005 treatment on the Ca^2+^ transients. qRT-PCR results showed that ZLN005-treated hESC-CMs exhibited higher expression of genes encoding for proteins involved in Ca^2+^ handling (ATP2A2, RYR2, PLN) (Figure 6A). To determine whether the changes in these genes affected hESC-CMs Ca^2+^ transients, spontaneous intracellular calcium ([Ca^2+^]_i_) transients in the hESC-CMs were recorded with Fura-2AM dye. Representative traces of Ca^2+^ influx for the two groups are shown in Figure 6B. While the amplitude of Ca^2+^ transient remained unchanged after treatment with ZLN005, the maximal velocity (V_max_) of Ca^2+^ upstroke (2.99 ± 0.64 vs. 7.43 ± 1.43 F/F0/sec, *P*= 0.004) and V_max_ decay (2.99 ± 0.64 vs. 7.43 ± 1.43 F/F0/sec, *P*=0.004) were significantly higher in the ZLN005-treated CMs (Figure 6E, 6G). Moreover, time to peak [Ca^2+^]_i_ (0.52 ± 0.08 vs. 0.25 ± 0.06 sec, *P*=0.0002) and decay time constant (1.45 ± 0.13 vs. 0.73 ± 0.07 sec, *P*<0.0001) in the ZLN005-treated CMs were significantly shorter than the control cells (Figure 6D, 6F). Taken together, these results provide evidence for the enhanced Ca^2+^ transient kinetics in the ZLN005-treated cells.

## DISCUSSION

hPSC-CMs are promising candidates for drug development, human disease modeling, and cardiac toxicity screening *in vitro*. In order to promote the practical application of hPSC-CMs, numerous studies have aimed to enhance hPSC-CMs’ maturation using chemical, genetic, and biomechanical approaches. Here, we focus on the bioenergetics of hESC-derived cardiomyocytes and functional impact of its manipulation. We demonstrated that increasing PGC-1α strongly stimulated mitochondrial biogenesis and promoted cardiomyocyte maturation. For the first time, our results revealed that the small molecule ZLN005 helped facilitate hPSC-CMs maturation by: 1) promoting maturation of mitochondrial biogenesis and energy metabolism; 2) enhancing structural maturation, including anisotropy, and sarcomere length; 3) increasing expression of CX43 and improving electrical activity; and 4) improving Ca^2+^ handling and electrophysiological properties. The application of ZLN005 both increased the electronic excitation and metabolic output, which led to better coupling of the energy supply with the electrical and contractile demand.

Increasing evidence has emphasized the importance of mitochondrial oxidative metabolism in the functional maturation of hPSC-CMs [6, 17, 18, 23]. PGC1α is a transcriptional co-regulator that regulates mitochondrial oxidative metabolism by activating genes involved in the OXPHOS and mitochondrial biosynthesis [14]. In addition, the perinatal period is a critical window for maturation of CMs [14, 43]. Our data show that the expression of PGC-1α was greatly upregulated at day 7 (spontaneous contraction began) of cardiac induction (Figure 1A), consistent with previous reports [44]. Multiple studies have shown that relatively mature hPSC-CMs exhibited a higher level of PGC-1α mRNA, which accompanied a higher mtDNA content and mitochondrial mass [18, 43]. Overexpression of PGC-1α in cardiomyocytes would induce hundreds of genes that encode for key enzymes in mitochondrial biogenesis and energy metabolism [24].

Since early-stage hPSC-CMs has been shown to display significant plasticity [24], we postulated that PGC-1α would regulate mitochondrial biogenesis program during the early maturation stage of hPSC-CMs. To efficiently and economically manipulate PGC-1α activity, ZLN005 was applied during the early time window. Consistent with previous studies, treatment of ZLN005 induced the upregulation of the PGC-1α gene and protein levels in hESC-CMs. As reported previously, ZLN005 increases the expression of PGC-1α and its downstream genes by promoting its transcriptional activity, which is dependent on the myocyte enhancer factor 2 (MEF2) binding site at the promoter region of the PGC-1α gene [37]. Moreover, we found that ZLN005 treatment also increased expression of the coactivated gene, estrogen-related receptor γ (ERRγ) (Figure 1D), which regulates mitochondrial metabolism, such as the tricarboxylic acid (TCA) cycle and oxidative phosphorylation (OXPHOS) [45]. This observation is complementary with our functional data, which showed that the resting OCR and ATP production are elevated in ZLN005-treated cells. It has been identified that the PCG-1α expression stimulated by ZLN005 in L6 myotubes is dependent on the AMPK pathway [41]. Moreover, activated AMPK enhances glucose/fatty uptake and mitochondrial metabolism by phosphorylating GLUT4/CD36 and PGC-1α/SIRT, respectively, which ultimately increases ATP production in skeletal muscle cells [46–48]. Certainly, this possibility should be further investigated in hPSC-CMs.

Although glycolysis is a major source of energy for cardiomyocytes during early cardiac development [21, 49], fatty acid oxidation becomes the primary source for fully matured cardiomyocytes [22, 50]. Indeed, dramatic shifts in energy metabolism during the cardiac fetal-to-adult transition have been reported [15]. The switch from glycolysis to fatty acid oxidation in cardiac metabolism is mainly mediated by the PGC-1α axis [51]. Similarly, the majority of energy production occurs by glycolysis in hPSC-CMs, while adult CMs use mostly oxidative metabolism [16, 52]. Upregulation of PGC-1α in cultured cardiomyocytes induced expression of mitochondrial genes involved in the multiple mitochondrial energy production and energy transduction pathways [50]. In this study, after treatment with ZLN005, several downstream transcription factors (TFAM, NRF1) of PGC-1α that promote mitochondrial biogenesis and metabolism were increased. PGC-1α transactivates NRF1, which subsequently activates TFAM, and consequently regulates mtDNA transcription and replication [53, 54]. As expected, the mtDNA copy number in ZLN005-treated CMs was higher than in controls. Moreover, a previous study reported that ZLN005-treatment increased the protein expression levels of OXPHOS complexes I, II, III and IV in bovine hepatocytes [55]. Consistent with these prior findings, our data show that the expression of several genes that encode for respiratory chain subunits and other genes that are responsible for mitochondrial biogenesis were significantly upregulated in ZLN005-treated CMs.

Additionally, a recent report indicates that shifting cultured hPSC-CMs’ metabolism could improve its metabolic and functional maturation [17]. Here, an increase in resting OCR and ATP productions were observed in ZLN005-treated CMs, which was most likely a result of increased mitochondrial content or increased mitochondrial activity [56]. Mitochondrial bioenergetics and ATP production provide key insights into the maturity of cardiomyocytes, since proper mitochondrial function is more crucial in adult and mature cardiomyocytes compared to neonatal and immature cardiomyocytes [16, 43]. Furthermore, our transmission electron microscopy results showed that ZLN005-treated hESC-CMs contained more mitochondria and exhibited better mitochondrial morphology and mitochondrial content compared with the controls. Hence, changes in mitochondrial content are critical components of cardiomyocyte maturation during development [8, 15, 21], which might contribute to an overall increase in mitochondrial oxidative phosphorylation. These data suggest that ZLN005 treatment increased PGC-1α and downstream gene expression and improved mitochondrial biogenesis and metabolic maturation in hESC-CMs. Compared to adult cardiomyocytes, immature cardiomyocytes exhibited smaller Ca^2+^ transient amplitudes and slower rises and decay kinetics. The electromechanical activity of cardiomyocytes is highly dependent on ATP produced by oxidative phosphorylation [57, 58].

Recent studies reported [11, 17, 18] that shifting cardiomyocyte metabolism to oxidative pathways promoted cellular maturation, which affected sarcomere lengths and Ca^2+^ transient kinetics. In particular, PGC-1α plays a key role in the glucocorticoid-induced maturation of fetal cardiomyocyte structure, oxygen consumption, ion channel expression, and Ca^2+^ handling [59, 60]. In line with this, we observed that ZLN005 treatment significantly increased the expression of representative of structural genes (MYL3, MYL4, MYH7, TNNT2) in hESC-CMs. Consistent with the increased gene expression, the sarcomere lengths and circularity were improved in ZLN-treated CMs. These findings suggest that ZLN005 treatment promoted hPSC-CMs’ morphology maturation, most likely due to a PGC-1α-driven increase in mitochondrial capacity [59]. Furthermore, Ca^2+^ imaging analysis showed that ZLN005-treated hESC-CMs exhibited significant increases in the upstroke rate and decay rates compared with controls, indicating more effective Ca^2+^ handling. In addition, ZLN005-treated hESC-CM exhibited a more negative resting membrane potential compared to the control group. Connexin 43 (CX43), as the major connexin protein in ventricular cardiomyocytes, brings about electrical excitability and mechanical coupling between cardiac cells [61–63]. It has been identified that substantial deficiencies in cell-cell coupling of immature hPSC-CMs may result from low CX43 membrane expression levels [16, 64]. The present study is the first to demonstrate that the expression of CX43 is significantly upregulated in the ZLN005-treated hESC-CMs. Importantly, hESC-CMs that were treated with ZLN005 exhibited more homogeneous, spontaneous beating rhythm and electrical propagation (supplementary movies). During cardiomyocyte maturation, CX43 becomes concentrated toward the cell-cell junction, which contributes to the final cell coupling and conduction velocity. A recent report demonstrated that increased CX43 expression improved cell coupling and excitability of iPSC-CMs [65]. Indeed, our data shows that ZLN005-treated CMs exhibited more uniform electrical propagation and higher electrical excitability, consistent with the increased expression of CX43 gap junctions. The results indicated that ZLN005 treatment improves cell-cell communication in hESC-CMs. During cardiac development, PGC-1α plays a key role in controlling the complex regulatory network in cardiac energetics. We sought to determine whether ZLN005 (a small-molecule transcriptional regulator of PGC-1α) promotes the maturation of hESC-CMs. As expected, ZLN005 significantly increased the expression of PGC-1α and improved mitochondrial biogenesis and metabolic maturation. In addition to stimulating mitochondrial development, ZLN005 treatment also promoted multiple aspects of hPSC-CM maturation.

In summary, we have shown that the small molecule compound ZLN005 is a valuable candidate for hPSC-CM maturation. ZLN005 treatment not only increased the expression of PGC-1α in hESC-CM, but also induced a multitude of target genes that could potentially promote maturation. Importantly, compared with the control hESC-CMs, ZLN005-treated hESC-CMs exhibited a more mature phenotype in various aspects than the control group, including mitochondrial oxidative metabolism, cellular structural, intercellular mechanical coupling, and Ca^2+^ handling. This study provides a simple and useful approach that will help improve the applications for cardiac disease modeling, drug screening, and regenerative medicine.

## MATERIALS AND METHODS

### Human ESCs culture and cardiomyocyte differentiation

Briefly, hESCs (H7, WiCell) were cultured in mTeSR1 media (Stem Cell Technologies, Vancouver, Canada) on Matrigel coated dishes (hESC qualified; Corning, New York, NY, USA) based method as previously described [66–68]. Cardiomyocyte differentiation was followed the protocol reported previously [69], hESCs with density of 80% confluence were digested into single cells by Accutase (Cat No. 07920, StemCell Technologies) and seeded onto Matrigel-coated dishes at a density of 3×10^4^ cells/cm^2^ in mTeSR1 for 3 days. When the cell confluence reached 90%, the media was changed from mTeSR1 to chemically defined medium (CDM), which consisted of RPMI 1640 medium (Cat No. 11875, Life Technologies), 213 μg/ml L-ascorbic acid 2-phosphate (Cat No. A8960, Sigma-Aldrich), and 200 μg/ml Bovine Serum Albumin (Cat No. A1933, Sigma-Aldrich) modified from previously reported. For day 0 to day 2, medium was supplemented with 6 μM CHIR99021 (Cat No. Axon 1386, Axon Medchem, Groningen, Netherlands). From day 2 to day 4, the medium was supplemented with 5 μM IWR-1 (Cat No. 681669, Merck). From day 4, the medium was changed to CDM every other day. Beating clusters of CMs were observed at day 7. The CMs were then homogenized at differentiation day 8 by trypsinization and re-seeding and treated with 10 μM ZLN005 (MCE, NJ, USA) at differentiation day 10 for 2 days as previously reported [41, 55].

### Immunostaining

The cells were fixed in 4% paraformaldehyde for 10 minutes and then permeabilized in 0.4% Triton X-100 for 6 minutes. The fixed cells were blocked with 5% normal goat serum for 1 hour at room temperature. Samples were incubated overnight at 4°C with following primary antibodies: mouse polyclonal anti-α-ACTININ (1:400, Sigma), rabbit polyclonal anti-connexin-43 (0.5μg/ml, Boster Biological). The samples were rinsed with PBS and stained for 1 hour with the following secondary antibodies: goat anti-mouse IgG Alexa Fluor488, goat anti-rabbit IgG Alexa Fluor594 (all from Jackson Immuno Research Laboratories, West Grove, PA, 1:1,000). All samples were counterstained with Hoechst 3342 (1μg/ml, Sigma-Aldrich) for 3 min. A Zeiss Observer microscope was used for slide observation and image capture. Sarcomere lengths were measured using Image J software. We analyzed the hESC-CM sarcomere length (measured by the distance between Z-disks) and the cell circularity index (Circularity = 4π·Area / Perimeter^2^) using previously published methods [43, 70, 71].

### Calcium (Ca^2+^) imaging

Cardiomyocytes were loaded with Fura-2 AM (Molecular Probes Inc, Eugene, OR) as previously described [43]. Briefly, cells were incubated with 5 μM Fura 2 AM in Tyrodes Solution (140mM NaCl, 5mM KCl, 1mM MgCl_2_, 1.8mM CaCl_2,_ 10mM glucose and 10mM HEPES, adjust pH 7.4 with NaOH) for 5 min at 37°C and then washed three times with PBS. The spontaneous calcium transient of single cardiomyocyte was recorded with a 40× Olympus objective using a Nikon Eclipse T5100 fitted with an IonOptix video microscopy system (Ionoptix Corporation, Milton, MA, USA). The experiments were done with culturing medium containing 15 mM HEPES at 35°C.

### Patch clamp for action potential recordings

Spontaneous action potentials (APs) of cardiomyocytes were recorded using a HEKA EPC-10 amplifier (HEKA, Lambrecht, Germany) as previously reported in detail [43]. Patch pipettes with resistances of 2-4 MΩ were prepared from 1.5-mm thin-walled borosilicate glass tubes using a Sutter micropipette puller P-97. External Tyrodes bath solution contained (in mmol/L): NaCl 140, KCl 5, MgCl_2_ 1, CaCl_2_ 1.8, KH_2_PO_4_ 0.4, glucose 10, HEPES 5, adjusted to pH 7.4 with NaOH. The pipette solution contained (in mmol/L): K^+^ aspartate 110, KCl 20, MgCl_2_ 1, Na-GTP 0.1, Mg-ATP 5, Na_2_-phosphocreatine 5, EGTA 5, HEPES 10, adjusted pH to 7.3 with KOH. All experiments were performed at 35°C, and all data were analyzed using Patchmaster (HEKA) and IgorPro software.

### Quantitative PCR and mitochondrial DNA content

Total RNA was extracted with RNAiso Plus Kit (TAKARA) and quantified using NanoDrop (Thermo Scientific). Reverse transcription was performed using the RevertAid First Strand cDNA Synthesis Kit (Thermo Scientific) following the manufacturer’s instructions. Quantitative PCR was performed on a Bio-Rad iCycler iQ (Bio-Rad) where each well contained a reaction mix totaling 20 μl, with 10μl of SYBR Premix Ex Taq (TAKARA), 5μl of cDNA (10 ng), and 5μl of primers (400 nmol/l each). Each assay was repeated three times, and the relative mRNA levels were normalized using GAPDH as the housekeeping gene.

The mitochondrial DNA Content was quantified as described previously [18]. Genomic and mitochondrial DNA was extracted using Genomic DNA Extraction Kit (TaKaRa). Relative mtDNA content was measured by quantitative real-time PCR (Applied Biosystems 7900) using primers for mitochondrially encoded NADH dehydrogenase (mt-ND1 or mt-ND2); values were normalized to the nuclear gene ACTB. Table 1 shows the primers used.

**Table 1:** The used primers in the study.

**Gene name**	**Primer sequence (5’-3’)**
PGC-1α	F: ACGAAGCAGACAAGACCGG
	R: GATTGCGTGCCATCCCAAG
PGC-1β	F: GAAGACATGCAGGCGATGGT
	R: CCAGACGGTAGGGTTTGCTG
ERRα	F: GGGAACTTCTGGCTCAAGACG
	R: CTGCGATCCTTACCTCCTCCA
ERRβ	F: GAACTTCCTCGGGCCACACT
	R: ACAGAGGGAGGGCAGAGTCT
ERRγ	F: GGCCATCAGAACGGACTTGAC
	R: CTTGGGCATCGAGTTGAGCAT
MFN1	F: GAAGGCAGGGTGACAGTGTG
	R: CTGCTGCAACCTGGTGTCTC
HADHA	F: GTGGGTGTGGAGGTTATGCA
	R: TCCCACCATTCTGTCAAGGC
SLC25A20	F: GGCAAATCCTCCTGACAGCT
	R: GATGATCCGAGCCTTCCCAG
ECHS1	F: CCAAACGTCACACTCCGGTA
	R: CCTGGAATGAGCAGAGCCAA
CKMT2	F: GCCACTTCAGCCTCTTTCAAAG
	R: ACTGCATCACAACTAGACCCTG
CS	F: CAGCACCAGGGAACAAGCAT
	R: GACTGTAGGGTGAGGGTGCT
NDUFA4	F: CAAACACTAGGCGAGGCAGG
	R: GGTGGCTAGGTCGGTTCTCT
ATP5FB	F: GAAGTCAGGCCTCTTTCCGC
	R: TCTTCGACCCAGCTCTGTCC
SDHB	F: GGTTCCAGCAGTCATTCGC
	R: TGCAGTGCTTTGGTTTGGC
ATP5PD	F: CCACAGGACCAGAGCGAAAT
	R: GTTGAGACTGCAGGGGTGAA
ACAA2	F: GGAGGTGTGCTGTAATGGCT
	R: CCACCACTCCCAGCCAATAG
DNM1L	F: GGGACAGGAGAGGGAAGGAT
	R: AGCCTCAACAGAGCGATTCC
GABPA	F: CTGGGAGCAACTTACCTGGG
	R: CATCCCATGAGCAGCCCTAG
TFAM	F: GGATCCCAGGAAGTTGAGGC
	R: CGGAATGATGGCGTGCAATT
OPA1	F: CTGGTTCAGTCTGAGGGCTG
	R: TGCCAGCACTTCTCCAGATG
MYL3	F: GGCCAACTCCAGGTGCATAT
	R: CCATCGGTGAAGTCTTGCCT
MYL4	F: GCTCTCACAGTGACAGGCC
	R: GATGTCTCCCTGGCCTCCAC
MYH7	F: GGTGGACATGGATGGAGCAA
	R: CTCTTGCAGACAGTGACCGT
MYH6	F: GGTGGAGGGTGTGTGTGTGA
	R: GCTACCGTCGATCAGCCTGT
TNNT2	F: CGTCAATGCGGTGGACATG
	R: CGCCCAGCATCTCCATCTC
TNNI3	F: CCACTGCACCTGGACCTTCA
	R: CGAGAAGGTGAGTGTGGGCT
ATP2A2	F: GGAGTGCTTAGCCCTGTGTT
	R: CACGGGCTTAACTGGATGGT
PLN	F: CCTGGCCAACTCCCTCCTTT
	R: CACCTGGCCGAGTAGTCACC
RYR2	F: CTGTCGGATCCTGTCACGCT
	R: GCAGTACTCGCCCGTCATCT
CX43	F: GGTGACTGGAGCGCCTTAG
	R: GCGCACATGAGAGATTGGGA
GAPDH	F: GGAGCGAGATCCCTCCAAAAT
	R: GGCTGTTGTCATACTTCTCATGG
MT-ND1	F: ATGGCCAACCTCCTACTCCTCATT
	R: TTATGGCGTCAGCGAAGGGTTGTA
MT-ND2	F: CATCTTTGCAGGCACACTCATCA
	R: ATTATGGATGCGGTTGCTTGCGTG
ACTB	F: CATGTACGTTGCTATCCAGGC
	R: CTCCTTAATGTCACGCACGAT

### Western blots

Cardiomyocytes were homogenized in ice-cold RIPA lysis buffer (Beyotime) containing protease inhibitors (BioTool), ultrasonicated for 10 s, and centrifuged (12000 rpm) at 4 °C for 15min. The supernatants were collected for estimating protein concentrations with an Enhanced BCA Protein Assay Kit (Beyotime). Protein abundance was measured by western blotting and normalized against GAPDH as described previously [72]. The primary antibodies were as follows: anti-PGC-1α antibody (1:500, Boster biological), Connexin 43 antibody (1:400, Boster biological), and anti-GAPDH antibody (1:5000, Abcam). The secondary antibody was the corresponding horseradish-peroxidase-coupled rabbit anti-mouse or mouse anti-goat antibody (1:2500, Beyotime). For quantification of immunoreactivities, western blots were visualized using Tanon-5200 Chemiluminescent Imaging System (Tanon) and subjected to densitometric analysis by using AlphaView Software.

### ATP assay

Cellular ATP content was determined using an Enhanced ATP Assay Kit (Beyotime) according to the manufacturer’s instructions. Briefly, cells were lysed with ATP lysis buffer and centrifuged at 12,000 g for 5 min at 4°C. The supernatant was collected and mixed with the assay buffer at room temperature. Luminance (RLU) was assayed in the CLARIOstar microplate reader (BMG LABTECH, Germany). The luminescence data were normalized by the sample protein concentrations.

### Transmission electron microscopy (TEM)

For TEM studies, cells were fixed with 4% glutaraldehyde solution (pH 7.2) for 2 hours and further fixed with 1% OsO4 in Sorenson’s buffer for 1 hour. After dehydration, the cells were embedded in Epon 812. Ultrathin sections (60 nm) were poststained with uranyl acetate and lead citrate. Sections were examined on a transmission electron microscope (TECNAL G2 Spirit Biotwin) operating at a voltage of 100 kV. Images were acquired at random locations throughout the samples.

### Measurement of cellular respiration

Oxygen consumption rate experiments were performed by the CLARIOstar microplate reader (BMG LABTECH, Germany) as previously described [72, 73]. In brief, CMs were plated in 96-well assay plates with a density of 30,000 per well in Culture medium. Replace seeding medium with 100 μl of culture media containing MitoXpress Intra probe at 10μg/ml after 24 hours culture. Then 100μL of pre-warmed mineral oil was overlaid in each well to create a seal. The plates were immediately read on the microplate reader according to manufacture protocol for 2 hours. A ratiometric analysis was performed to determine changes in the Lifetime fluorescence signal. Lifetime slopes were calculated using each Lifetime measurement over time. These conversions were performed using the MARS data analysis software.

### Multi electrode array (MEA)

Standard 60-electrode MEA probes (Multi Channel Systems, Germany) were plasma-cleaned and coated with Matrigel for 1 h at 37°C. Monolayer hESC-CMs were dissociated and re-plated on MEA probes. Extracellular recording was performed using a MEA2100-System (Multi Channel Systems, Germany). Output signals were digitized at 10 kHz. Recordings were performed between day 14 and day 21 after the cardiac differentiation. During recordings, temperature was kept at 37°C. Data were recorded using Cardio2D (Multi Channel Systems, Germany) and analyzed off-line with Cardio2D+ (Multi Channel Systems, Germany).

### Statistical analysis

All of the data were shown as mean ± SEM and statistical analyses were performed using Microsoft GraphPad Prism software (LA Jolla, CA). Student’s t test was applied for the comparisons between ZLN005 treatment and control group. One-way analysis of variance (ANOVA) followed with Bonferroni's multiple comparisons was applied for the PGC-1α and PGC-1β expression during cardiomyocyte differentiation day 0 to day 30. Two-way ANOVA was applied with Tukey's multiple comparison for the quantitative analysis of PGC1α protein expression levels at differentiation day 15 and day 30 after ZLN005 treatment. P < 0.05 was regarded as statistically significant.

## Supplementary Material

Supplementary Figure 1

Supplementary Movie 1

Supplementary Movie 2

Supplementary Movie 3

Supplementary Movie 4
