# Supplementary material for: PGC-1α activator ZLN005 promotes maturation of cardiomyocytes derived from human embryonic stem cells
Source: Aging (Albany NY). 2020 Apr 28;12(8):7411–30. doi: 10.18632/aging.103088 (PMC7202542; doi:10.18632/aging.103088)
Supplement: Supplementary Figure 1 [file aging-12-103088-s001..pdf]

SUPPLEMENTARY FIGURE

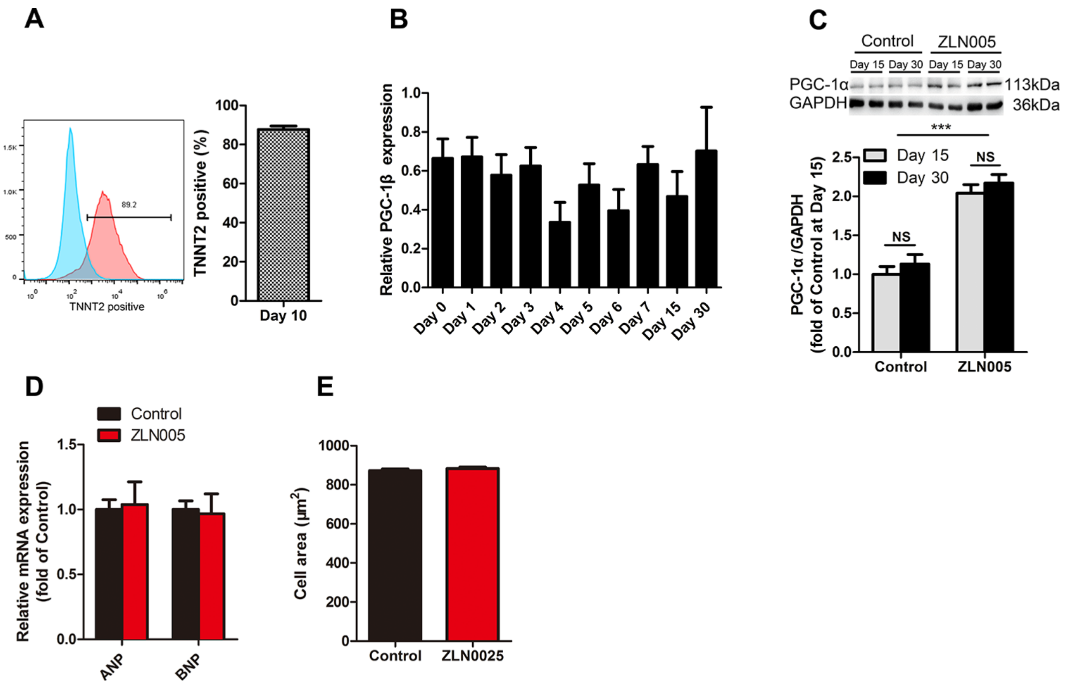

Supplementary Figure 1. (**A**) Cardiac differentiation efficiency measured by flow cytometry for cardiac troponin T (TNNT2) on day 10 cells (n=5). (**B**) The relative mRNA expression of PGC-1β during cardiomyocyte differentiation (n=7). (**C**) Effect of ZLN005 on PGC-1α protein expression (n=4). (**D**) qRT-PCR analysis of cardiac hypertrophy marker genes in hESC-CMs (n=7). (**E**) The cell area was not significantly changed in ZLN005-treated hESC-CMs (n=20-60 cells per condition).
